# Supplementary material for: Prognostic Biomarkers of Salvage Chemotherapy Following Nivolumab Treatment for Recurrent and/or Metastatic Head and Neck Squamous Cell Carcinoma
Source: Cancers (Basel). 2020 Aug 15;12(8):2299. doi: 10.3390/cancers12082299 (PMC7463840; doi:10.3390/cancers12082299)
Supplement: Supplementary file 1 [file cancers-12-02299-s001.pdf]

Supplementary Material: Prognostic Biomarkers of Salvage Chemotherapy Following Nivolumab Treatment for Recurrent and/or Metastatic Head and Neck Squamous Cell Carcinoma

Takahiro Wakasaki, Ryuji Yasumatsu, Muneyuki Masuda, Toranoshin Takeuchi, Tomomi Manako, Mioko Matsuo, Rina Jiromaru, Ryutaro Uchi, Noritaka Komune, Teppei Noda and Takashi Nakagawa

Table S1. Profiles of adverse events appearing during salvage chemotherapy following nivolumab.

Category	Number of Events(all *n* = 39)				Number of Events(PC *n* = 25)				Number of Events(S1 *n* = 14)			
	Total (*n*)	(%)	Grade 3/4 (*n*)	(%)	Total (*n*)	(%)	Grade 3/4 (*n*)	(%)	Total (*n*)	(%)	Grade 3/4 (*n*)	(%)
Any	32	82.1	17	43.6	24	96.0	16	64.0	8	57.1	1	2.4
Skin disorder	13	33.3	5	12.8	13	52.0	5	20.0	0	0.0	0	0.0
Perionychia	3	7.7	1	2.6	3	12.0	1	4.0	0	0.0	0	0.0
Gastrointestinal	2	5.1	0	0.0	1	4.0	0	0.0	1	7.1	0	0.0
Hepatobiliary	3	7.7	0	0.0	2	8.0	0	0.0	1	7.1	0	0.0
Interstitial pneumonitis	5	12.8	1	2.6	4	16.0	1	4.0	1	7.1	0	0.0
Fatigue	4	10.3	1	2.6	3	12.0	1	4.0	1	7.1	0	0.0
Fever	3	7.7	0	0.0	1	4.0	0	0.0	2	14.3	0	0.0
Peripheral neuropathy	1	2.6	0	0.0	1	4.0	0	0.0	0	0.0	0	0.0
Leukopenia	20	51.3	16	41.0	16	64.0	15	60.0	4	28.6	1	2.4
Neutropenia	15	38.5	10	25.6	14	56.0	10	40.0	1	7.1	0	0.0
Hypomagnesia	4	10.3	3	7.7	4	16.0	3	12.0	0	0.0	0	0.0
Others	6	15.4	2	5.1	3	12.0	2	8.0	3	21.4	0	0.0

AEs, adverse events; S1, tegafur-gimestat-otastat potassium; SCT, salvage chemotherapy.

Table S2. Summary of the previous definitive therapies and the administration lines of SCT.

Pt	SCT	Gender	Age	Primary Site	Previous Definitive Therapies	Administration Lines of SCT
1	PC	F	75	Oral	Ope, RT	4
2	PC	M	66	Oral	IC, Ope, CRT	2
3	PC	F	33	Oral	Ope, CRT	2
4	PC	M	60	Oral	Ope, RT	3
5	PC	M	63	Oral	IC, Ope, CRT	3
6	PC	M	71	Oral	Ope, CRT	2
7	PC	F	77	Oral	Ope, RT	3
8	PC	M	56	Nasopharynx	IC + CRT	2
9	PC	M	51	Nasopharynx	CRT	2
10	PC	M	74	Oropharynx	CRT	2
11	PC	M	47	Oropharynx	Ope, CRT	2
12	PC	M	62	Oropharynx	CRT	2
13	PC	M	63	Hypopharynx	IC, CRT	3
14	PC	M	65	Hypopharynx	CRT	2
15	PC	M	75	Hypopharynx	Ope, CRT	2
16	PC	M	54	Hypopharynx	CRT, Ope	2
17	PC	M	68	Hypopharynx	CRT	2
18	PC	M	64	Sinonasal tract	CRT	3
19	PC	M	73	Sinonasal tract	IC, Ope, CRT	2
20	PC	M	69	Sinonasal tract	IC, Ope, CRT	2
21	PC	M	66	Sinonasal tract	Ope, CRT	2
22	PC	M	56	Larynx	Ope, CRT	3
23	PC	M	69	Larynx	Ope	3
24	PC	F	45	Others	Ope	2
25	PC	F	78	Others	Ope, CRT	3
26	S1	M	71	Oral	Ope, CRT	2
27	S1	M	64	Oral	Ope, CRT	2
28	S1	F	40	Nasopharynx	IC, CRT	5
29	S1	M	59	Nasopharynx	None	3
30	S1	M	67	Oropharynx	CRT	3
31	S1	M	71	Oropharynx	IC, CRT, Ope	3
32	S1	M	59	Oropharynx	CRT, Ope	2
33	S1	M	68	Oropharynx	Ope, CRT	4

Table S2. *cont.*

Pt	SCT	Gender	Age	Primary Site	Previous Definitive Therapies	Administration Lines of SCT
34	S1	M	66	Hypopharynx	None	3
35	S1	M	69	Hypopharynx	IC, Ope, CRT	2
36	S1	M	73	Sinonasal tract	IC,CRT	4
37	S1	F	47	Sinonasal tract	Ope, CRT	3
38	S1	M	75	Sinonasal tract	IC, Ope, CRT	2
39	S1	M	64	Others	IC, RT	3

Pt, patient; SCT, salvage chemotherapy; Ope, operation; IC, induction chemotherapy; CRT, chemoradiotherapy.
